# Mesoporous Bioactive Glasses Incorporated into an Injectable Thermosensitive Hydrogel for Sustained Co-Release of Sr^2+^ Ions and *N*-Acetylcysteine

**DOI:** 10.3390/pharmaceutics14091890

**Published:** 2022-09-07

**Authors:** Carlotta Pontremoli, Monica Boffito, Rossella Laurano, Giorgio Iviglia, Elisa Torre, Clara Cassinelli, Marco Morra, Gianluca Ciardelli, Chiara Vitale-Brovarone, Sonia Fiorilli

**Affiliations:** 1 Department of Applied Science and Technology, Politecnico di Torino, Corso Duca degli Abruzzi 24, 10129 Torino, Italy; 2 Department of Chemistry, NIS Interdepartmental and INSTM Reference Centre, University of Torino, Via Giuria 7, 10125 Torino, Italy; 3 Department of Mechanical and Aerospace Engineering, Politecnico di Torino, Corso Duca degli Abruzzi 24, 10129 Torino, Italy; 4 Nobil Bio Ricerche Srl, Via Valcastellana 26, 14037 Portacomaro, Italy

**Keywords:** mesoporous bioactive glasses, injectable hydrogels, *N*-acetylcysteine, strontium ions, co-release, delayed bone regeneration

## Abstract

An injectable delivery platform for promoting delayed bone healing has been developed by combining a thermosensitive polyurethane-based hydrogel with strontium-substituted mesoporous bioactive glasses (MBG_Sr) for the long-term and localized co-delivery of pro-osteogenic Sr^2+^ ions and an osteogenesis-enhancing molecule, *N*-Acetylcysteine (NAC). The incorporation of MBG_Sr microparticles, with a final concentration of 20 mg/mL, did not alter the overall properties of the thermosensitive hydrogel, in terms of sol-to-gel transition at a physiological-like temperature, gelation time, injectability and stability in aqueous environment at 37 °C. In particular, the hydrogel formulations (15% *w/v* polymer concentration) showed fast gelation in physiological conditions (1 mL underwent complete sol-to-gel transition within 3–5 min at 37 °C) and injectability in a wide range of temperatures (5–37 °C) through different needles (inner diameter in the range 0.4–1.6 mm). In addition, the MBG_Sr embedded into the hydrogel retained their full biocompatibility, and the released concentration of Sr^2+^ ions were effective in promoting the overexpression of pro-osteogenic genes from SAOS2 osteoblast-like cells. Finally, when incorporated into the hydrogel, the MBG_Sr loaded with NAC maintained their release properties, showing a sustained ion/drug co-delivery along 7 days, at variance with the MBG particles as such, showing a strong burst release in the first hours of soaking.

## 1. Introduction

Bone is one of the few tissues able to completely heal without the formation of a fibrous scar [1]. Despite this fascinating ability to heal, regenerate and self-repair, in 5–10% of bone defects, especially those caused by trauma, cancer or concomitant diseases, the fracture healing process fails under the conventional treatments, leading to delayed healing or even to non-healing fractures [2]. Since non-healing bone defects have been continuously growing over the last decades, great efforts have been dedicated to the design of advanced and multifunctional biomaterials able to boost the bone repair process by providing morphological, chemical and biological cues [3,4,5]. For this purpose, mesoporous bioactive glasses (MBGs) represent one of the most promising and versatile biomaterials, since they combine the excellent bioactivity typical of bioactive glasses with the release of therapeutic agents able to provide specific biological effects. In addition, the mesopore size and surface chemistry of MBGs can be easily tailored, allowing the loading of a wide range of therapeutic molecules, including small drug molecules, and biomolecules (e.g., growth factors, peptides) to promote personalized therapeutic solutions. Several authors have further widened the therapeutic functionalities by enriching the MBG composition with specific metallic ions (i.e., Sr^2+^, Cu^2+^, Ag^+^, Ce^3+^) [6,7,8,9,10,11,12,13,14] able to impart several different biological effects, e.g., pro-osteogenic, anti-oxidant, pro-angiogenic abilities. Moreover, inorganic ions are involved in several cellular functions, inducing secondary signals by activating ion channels or interacting with other ions or with macromolecules [15].

Compared to the use of growth factors, recombinant proteins or other genetic engineering approaches, which are highly expensive and associated with a higher risk of side effects and short shelf life, the incorporation of therapeutic concentrations of inorganic ions presents several undoubted advantages, and it has recently emerged as a promising strategy to promote tissue regeneration in compromised clinical cases [13,16,17].

With this perspective, the biological response to Sr-substituted MBGs has been recently evaluated by the authors in terms of cell viability, inflammatory response, and anti-osteoclastogenesis activity [6,9,18]. In particular, the monitoring of the receptor activator of nuclear factor-κB ligand (RANKL) and osteoprotegerin (OPG) pathways revealed a RANKL/OPG ratio in favor of OPG induced by the released strontium ions, thus proving the inhibition effect on osteoclast activity and the beneficial role for bone regenerative purposes.

In order to further boost the bone regenerative potential, several authors have explored the loading of specific drugs into the porous structure of MBGs in order to attain multiple synergistic effects by ion/drug co-delivery [13,19].

Despite their remarkable properties, to date, some drawbacks still limit MBGs’ clinical translation, among which is the strong burst release of incorporated ions/drugs once in contact with body fluids, as well as the shortcomings associated with the administration in the form of powder. Since MBGs alone are not exploitable as long-term drug delivery systems for orthopedic applications, their combination with polymers or other vehicle phases able to modulate the release kinetics and/or act as a depot at the pathological site would greatly enlarge the clinical potential of these multifunctional nanocarriers.

Among the possible vehicle phases, injectable hydrogels have been recently proposed as a very promising clinical platform, due to their ability to be applied through minimally invasive surgery and, upon gelling, to perfectly fill the bony defects. Specifically, temperature-responsive hydrogels have been proposed as drug delivery systems for tissue engineering applications.

For these reasons, the combination of MBGs with thermosensitive hydrogels, in which an aqueous polymeric solution undergoes a sol-to-gel transition in physiological conditions (37 °C), represents an attractive strategy to develop non-invasive devices, directly injectable at the clinical site and acting as a drug reservoir for sustained and localized release.

In this context, Pluronic^®^ F-127 (commercially known also as Poloxamer^®^ 407) aqueous solutions have gained much attention as cell and drug delivery systems thanks to their low toxicity, reverse thermal gelation, high drug-loading capabilities and ability to gel in physiological conditions at relatively low concentrations [20]. However, despite their excellent biocompatibility, Pluronic^®^ F-127-based hydrogels show an important drawback: their poor stability in an aqueous environment leads to the dissolution of the hydrogels within the first few minutes after injection, limiting their use as prolonged and sustained release devices. In order to overcome this limitation, the Pluronic^®^ F-127 chain can be extended by exploiting the chemistry of polyurethanes, which allows one to develop a versatile and flexible platform for different applications in the regenerative medicine and biomedical fields, in particular, as bioerodible/biodegradable scaffolds and delivery systems [7,21,22]. For this purpose, Boffito M. et al. [21] reported a procedure to produce a novel amphiphilic poly(ether urethane) (PEU) based on Poloxamer^®^ 407, which, in aqueous solutions, exhibited improved gelation ability, mechanical strength and stability compared to similar formulations based on the commercially available Poloxamer^®^ 407, as such.

**Figure 1:**
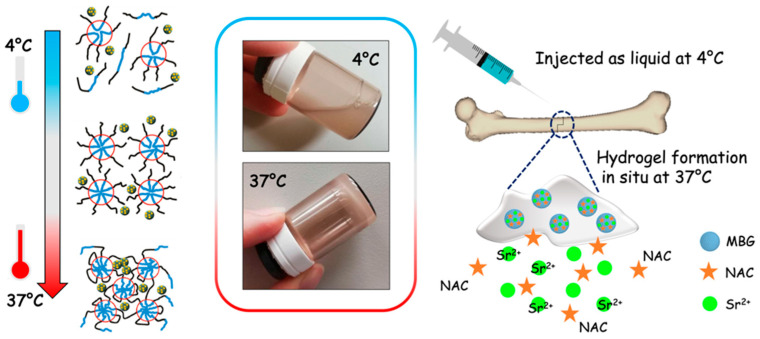
The thermo-responsive hydrogel as an injectable platform for the co-delivery of strontium ions and NAC from incorporated MBGs.

In this contribution, with the aim being to design an injectable platform for bone-related applications, the developed thermosensitive PEU-based hydrogel has been combined as a vehicle phase with MBG microparticles for the long-term and localized co-delivery of pro-osteogenic Sr^2+^ ions and an osteogenesis-enhancing molecule, N-Acetylcysteine (NAC), as schematically outlined in Figure 1.

The incorporation of NAC, an antioxidant derivative from the amino acid cysteine, was driven by its proven ability to induce the activation of osteoblastic differentiation [23,24]. Yamada et al. [23] demonstrated the bioactive role of NAC when administrated in the range of 2.5–10 mM in the increase of osteoblastic phenotypic expression, alkaline phosphatase (ALP) activity and extracellular matrix mineralization on rat osteoblastic culture derived from femur bone marrow. Moreover, the administration of a therapeutic amount of NAC is able to up-regulate the expression of bone-related genes (collagen I, osteopontin, osteocalcin, bone morphogenetic protein 2 (BMP-2)).

The obtained hydrogel–MBG system has been investigated in terms of sol-to-gel transition temperature and time, injectability and stability in an aqueous environment at 37 °C to mimic the real working conditions of the developed delivery platform. In addition, the biocompatibility and pro-osteogenic response has been evaluated to assess if the released Sr^2+^ ion concentration is able to impart anti-osteoclastogenesis activity, as was previously proven by the authors on Sr-containing MBGs alone [6,9].

Finally, the co-release profiles of Sr^2+^ ions and NAC from the MBGs incorporated into the hydrogel have been assessed and the obtained kinetics compared to those obtained for MBGs alone.

## 2. Materials and Methods

### 2.1. Synthesis of Sr-Substituted MBGs

#### 2.1.1. Materials

Double-distilled water (ddH_2_O), tetraethyl orthosilicate (TEOS), calcium nitrate tetrahydrate (Ca(NO_3_)_2_ × 4H_2_O, 99%), strontium chloride (SrCl_2_ 99%), Pluronic^®^ P123 (EO_20_PO_70_EO_20_, $\bar{M_{n}}$ = 5800 Da), *N*-acetylcysteine (>98% GC), Trizma^®^ base, primary standard and buffer, 99.9% (titration), were purchased from Sigma Aldrich (Milan, Italy) and used as received. All solvents were purchased from Sigma Aldrich (Milan, Italy) in analytical grade.

#### 2.1.2. Sr-Substituted MBGs

The micro-sized MBGs were produced by exploiting a water-based process under mild acidic conditions by using an aerosol-assisted spray-drying approach, following the procedure previously reported by the authors [6,9]. As reference for the biological response, MBGs without ions were produced, by following the procedure reported by Berkmann et al. [25]. MBGs with the 2% molar percentage of Strontium (Sr-substituted MBGs, molar ratio Sr/Ca/Si = 2/13/85, named hereafter as MBG_Sr) were prepared as follows: (Solution I) the non-ionic block copolymer Pluronic^®^ P123 (2.03 g) was dissolved in ddH_2_O (85 g). (Solution II) TEOS (10.73 g) was pre-hydrolyzed under acidic conditions using an aqueous HCl solution at pH = 2 (around 5 g) for 2 h to obtain a transparent solution. Solution II was then added dropwise into the Solution I and stirred for 30 min. After 30 min, strontium chloride (0.32 g) and calcium nitrate tetrahydrate (1.86 g) were added. The obtained solution was stirred for 15 min and then sprayed (Büchi, Mini Spray-Dryer B-290) using nitrogen as the atomizing gas, setting the following parameters: inlet temperature 220 °C, N_2_ pressure 60 mmHg and feed rate 5 mL min^−1^. The obtained powder was collected and calcined at 600 °C in air for 5 h at a heating rate of 1 °C min^−1^ using a FALC FM 8.2.

### 2.2. Synthesis and Characterization of the Hydrogel-Forming Material

#### 2.2.1. Materials

The commercially available triblock co-polymer Poloxamer^®^ 407 (P407, poly(ethylene oxide)-poly(propylene oxide)-poly(ethylene oxide), PEO-PPO-PEO, $\bar{M_{n}}$ = 12,600 Da) was purchased from Sigma Aldrich (Milan, Italy) and used as macrodiol for PEU synthesis after drying at 100 °C for 8 h and cooling at 40 °C under a vacuum (approx. 200 mbar). Furthermore, 1,6-hexamethylene diisocyanate (HDI) and N-Boc serinol were also purchased from Sigma Aldrich (Milan, Italy) and used as a diisocyanate and chain extender during PEU synthesis, respectively. Before use, HDI was distilled under reduced pressure to remove moisture and stabilizers, while N-Boc Serinol was stored in a desiccator under a vacuum. The organotin compound dibutyltin dilaurate (DBTDL) was purchased from Sigma Aldrich (Milan, Italy) and added in a catalytic amount to catalyze the PEU synthesis reaction. The synthesis was performed using 1,2-dichloroethane (DCE, Carlo Erba Reagents, Milan, Italy) as a solvent. Before the synthesis procedure, DCE was anhydrified over activated molecular sieves (4 Å, Sigma Aldrich, Milan, Italy, activation at 120 °C overnight) under nitrogen atmosphere overnight. All other solvents required for PEU synthesis were purchased from Carlo Erba Reagents (Milan, Italy) in analytical grade and used as received.

#### 2.2.2. Poly(ether urethane) Synthesis

PEU synthesis was performed through a two-step procedure under N_2_ flow, according to our already published protocol (Figure S1) [21,26]. Briefly, 60 g of P407 was first solubilized in 300 mL of anhydrous DCE (20% *w/v* concentration) in a three-neck round bottom flask under a nitrogen atmosphere. After system equilibration at 80 °C, HDI and DBTDL were added at a 2:1 molar ratio and 0.1% *w*/*w* with respect to P407, respectively. The addition of DBTDL initiated the pre-polymerization step of the synthesis, leading to the formation of isocyanate-terminated prepolymer chains after 150 min of reaction at 80 °C. The reaction mixture was then cooled down to 60 °C, and N-Boc Serinol previously solubilized in anhydrous DCE (3% *w*/*v*, 1:1 molar ratio with respect to P407) was added to start the chain extension step that lasted 90 min. Then, the system was cooled down to room temperature (RT), MeOH was added to stop the reaction through passivation of remaining isocyanate groups and the polymer was collected by precipitation in petroleum ether (volume four-times higher than the total volume of DCE used during the synthesis). Purification was conducted through solubilization of the polymer in DCE (30% *w*/*v*, 200 mL) and precipitation in a 98:2 *v/v* diethyl ether:MeOH mixture (volume five-times higher than the DCE volume used to dissolve the polymer). The synthesized poly(ether urethane) (acronym NHP) was finally collected through centrifugation (Hettich, MIKRO 220R) at 0 °C and 6000 rpm for 20 min and dried under the fume hood overnight. The as-synthesized NHP was then subjected to a Boc-deprotection reaction, leading to the exposure of free primary amines along the polymeric chains. The deprotection reaction was conducted in acidic conditions according to our recently optimized protocol (Figure S2) [27]. Briefly, 10 g of NHP were first solubilized in 225 mL of chloroform at 250 rpm and RT for 120 min. Then, 25 mL of trifluoroacetic acid (TFA) were added to the polymer solution (overall polymer concentration of 4% *w*/*v*, 90:10 *v/v* chloroform: TFA), and the deprotection reaction was carried out for 60 min. At the end of the reaction, the TFA/chloroform mixture was evaporated under reduced pressure using a Buchi Rotavapor Labortechnik AG, and the collected polymer was solubilized in 100 mL of chloroform and subjected to vacuum evaporation again. This washing procedure was repeated twice to completely move TFA residues away. The polymer was then solubilized in 200 mL of demineralized water overnight, dialyzed (cellulose membrane with 10–14kDa cut-off, Sigma Aldrich, Milan, Italy) against demineralized water for 48 h at 4 °C (3 complete water refreshes/day) and freeze-dried using a Martin Christ ALPHA 2-4 LSC instrument. The collected polymer after the Boc-deprotection reaction was stored under a vacuum at 4 °C until use and is referred to with the acronym SHP.

#### 2.2.3. Chemical Characterization of the Synthesized Poly(ether urethane)

The successful synthesis of a poly(ether urethane) bearing primary amino groups along its polymeric chains was proven by Attenuated Total Reflectance Fourier Transformed Infrared (ATR-FTIR) spectroscopy, Size Exclusion Chromatography (SEC) and the colorimetric Ninhydrin assay.

The ATR-FTIR spectra were registered through a Perkin Elmer Spectrum 100 (Waltham, MA, USA) instrument bearing an ATR accessory with a diamond crystal (UART KRS5). Analyses were conducted at RT, and the measured spectra resulted from the mean of 16 scans within the wavenumber range from 4000 to 600 cm^−1^. The spectra resolution was set at 4 cm^−1^. Spectra were recorded for P407, NHP and SHP to assess (i) the success of poly(ether urethane) synthesis, (ii) the absence of degradation ascribed to the acidic conditions established during the Boc-deprotection reaction and (iii) the complete drying of the polymer (i.e., complete removal of solvent residues). Spectra were analyzed and compared using the Perkin Elmer Spectrum software.

Chromatographic analyses were conducted using an Agilent Technologies 1200 Series (Santa Clara, CA, USA) bearing a Refractive Index (RI) detector and two Waters Styragel columns (HR1 and HR4) equilibrated at 55 °C. *N*,*N*-dimethylformamide (DMF, HPLC grade, Carlo Erba Reagents, Milano, Italy) added with LiBr (0.1% *w*/*v*, Sigma Aldrich, Milan, Italy) was used as an eluent at a flow rate of 0.5 mL min^−1^. Samples were prepared by solubilizing the polymer in the mobile phase at a 2 mg mL^−1^ concentration. Each sample was then filtered (poly(tetrafluoroethylene) membrane with 0.45 μm pores, Lab Logistic Group GmbH, Meckenheim, Germany) and a volume of 20 μL was injected into the mobile phase using an autosampler to start the chromatographic separation. The registered RI signal vs. elution time was then analyzed using the Agilent ChemStation software and a poly(methyl methacrylate)-based calibration (standards with peak molecular weight in the range from 4 to 200 kDa) to get the polymer molecular weight distribution, average molecular weight values (i.e., the Number Average Molecular Weight ($\bar{M_{n}}$) and the Weight Average Molecular Weight ($\bar{M_{w}}$)) and polydispersity index (D = $\bar{M_{w}}$/$\bar{M_{n}}$).

The colorimetric ninhydrin assay, also commercially known as the Kaiser test, was conducted to prove the successful deprotection of NHP and quantify the number of free primary amines exposed along SHP backbone. The Kaiser test kit used in this work was purchased from Sigma Aldrich (Milan, Italy), and the test was performed according to the supplier’s instructions. Briefly, PEU samples (10 mg) were weighed into glass test tubes, and the kit’s reagents were added according to this order: (i) phenol 80% in EtOH (75 μL), (ii) KCN in H_2_O/pyridine (100 μL) and (iii) ninhydrin 6% in EtOH (75 μL). Samples were then mixed with a vortex and heated at 120 °C for 5 min in the dark. Then, they were diluted with an EtOH/ddH_2_O 60:40 *v/v* solution and analyzed using a Perkin Elmer (Waltham, MA, USA) Lambda 365 UV-Vis spectrophotometer, being the main absorption peak of the formed complexes between -NH_2_ groups and ninhydrin molecules at 570 nm. The number of free primary amines was finally estimated by referring to a calibration curve based on glycine standards. The analyses were conducted in triplicate, and results are reported as mean ± standard deviation. NHP samples were also analyzed according to the same protocol, as the control condition.

### 2.3. Design of the Hydrogel-Based Platform for Sustained Sr^2+^ Ion Release

#### 2.3.1. Incorporation of MBG_Sr into SHP Hydrogel

SHP thermo-responsive hydrogels were prepared at a final polymeric concentration of 15% *w*/*v*, in accordance with previous knowledge acquired on formulations based on similar poly(ether urethane)s [27]. Purely PEU-based hydrogels (SHP) were obtained by solubilizing the required amount of SHP in a predefined volume of the physiological solution (0.9% NaCl) to achieve the selected final concentration of 15% *w*/*v*. Solubilization was conducted overnight at 4 °C to avoid undesired micellization and gelation phenomena hindering complete polymer dissolution. In order to encapsulate MBG_Sr particles within the hydrogels (SHP_MBG_Sr), the preparation protocol was modified as follows. Briefly, the SHP powder was first solubilized in a physiological solution (0.9% NaCl) at 18.75% *w/v* concentration overnight at 4 °C. Then, an MBG_Sr particle dispersion at 100 mg mL^−1^ concentration was prepared in a physiological solution (0.9% NaCl) and sonicated at 20% amplitude for 3 min (Sonics, Vibracells). An aliquot of such dispersion was added to the SHP solution kept at 4 °C to achieve final polymer and MBG concentrations of 15% *w/v* and 20 mg mL^−1^, respectively. The obtained formulation was then mixed with a vortex for 30 s to homogeneously disperse the inorganic phase within the polymeric solution.

#### 2.3.2. Characterization of SHP_MBG_Sr Hydrogel

The developed SHP_MBG_Sr hydrogel formulation was characterized in terms of gelation potential, injectability and swelling/dissolution behavior in an aqueous environment at 37 °C. In all these characterizations, SHP_MBG_Sr samples were compared with purely SHP hydrogels to assess whether MBG encapsulation could affect their properties.

A tube inverting test was conducted to qualitatively evaluate the gelation temperature of both SHP_MBG_Sr and SHP hydrogels and their gelation time at physiological temperature (i.e., 37 °C). To this aim, hydrogels (1 mL) were prepared in Bijou sample containers (Carlo Erba Reagent, Milan, Italy) to avoid potential differences ascribable to sample geometrical features. With the aim to estimate the sol-to-gel transition temperature, samples were subjected to a controlled temperature increase within the range of 5–70 °C at 1 °C/step. In each step, the samples were kept at the set temperature (error ± 0.1 °C) for 5 min and then inverted to assess their sol or gel state, by checking the presence or absence of flow along Bijou walls within 30 s of tube inversion. Differently, to measure the gelation time at physiological temperature, samples were incubated at 37 °C within an incubator (Memmert IF75) for predefined time intervals within the range of 1–10 min (i.e., from 1 to 10 min, 1 min increase/step), inverted at the end of each time frame, and lastly, gelation was assessed as previously described. Then, the samples were equilibrated at 4 °C for 8 min before further incubation at 37 °C for the next time frame considered, to ensure that at the beginning of each step, all the systems were in the sol state.

Injectability was qualitatively evaluated at three temperatures using commercially available needles of different diameters. Briefly, three batches of SHP and SHP_MBG_Sr hydrogels (3 mL) were first loaded in the sol state into 5 mL plastic syringes and then equilibrated at three different temperatures (5, 25 and 37 °C), resulting in sol, semi-gel and gel formulations. Then, injectability was tested by three different potential users through commercially available G22, G18 and G14 needles with a standard bevel (inner diameter of 0.413, 0.838 and 1.6 mm, respectively). Injectability, quick gelation and capability to retain the shape were also qualitatively tested ex vivo using a turkey thigh bone containing a manually induced defect in the diaphysis. In order to perform this test, the bone was equilibrated at 37 °C in an incubator, the formulation was colored by adding a catalytic amount of Toluidine Blue O dye (Sigma Aldrich, Milan, Italy) and injection was performed in the sol state using a G18 needle.

The SHP and SHP_MBG_Sr hydrogel swelling potential and stability in an aqueous environment were tested by incubating the samples in the presence of Tris HCl buffer solution (0.1 M, pH 7.4) at 37 °C. In detail, the hydrogels (1 mL) were prepared in Bijou sample containers as previously described, weighed (W_i_) and incubated (Memmert IF75) at 37 °C for 15 min to allow complete gelation. Then, 1 mL of Tris HCl buffer solution (previously equilibrated at 37 °C) was gently added on top of each sample and the test started. A complete Tris HCl buffer solution refresh was performed every two days. At predefined time points (1, 7 and 14 days), three samples of both SHP and SHP_MBG_Sr hydrogels were collected and weighed (W_f_) after removal of the residual Tris HCl buffer solution. The samples were also weighed (W_f-dried_) after freeze-drying. Moreover, reference samples (i.e., samples not incubated with Tris HCl buffer solution) were prepared, freeze-dried and weighed (W_i-dried_) to define the initial dried weight of both SHP and SHP_MBG_Sr formulations. The percentage of swelling (Swelling (%)) and dried weight loss (Weight Loss (%)) were finally estimated according to the equations reported by Boffito et al. [21]. The residual material collected upon freeze-drying was also analyzed by SEC to assess the occurrence of potential polymer chemical degradation during incubation in physiological-mimicking conditions. SEC analyses were conducted according to the protocol described in Section 2.2.3.

### 2.4. Biological Assessment of the SHP_MBG_Sr Hydrogel

The biological assessment was conducted in the not-contact method, where the SHP_MBG_Sr sample was placed in a Transwell^®^ membrane insert (<3 µm pore, SARSTEDT AG & Co., Numbrecht, Germany) to allow the transfer of the hydrogel extracts. In particular, the SHP_MBG_Sr formulation was prepared as previously described, casted in a Petri dish and incubated at 37 °C for 1h to allow the sol-to-gel transition. After 1h, the gelled formulation was cut to obtain pieces of 0.2 g each and put in a Transwell^®^ 24 well plate. The fibroblast cell line L929 was used to assess the biocompatibility of SHP_MBG_Sr, while osteoblast-like cells (SAOS2) were selected to investigate the pro-osteogenic response.

#### 2.4.1. Biocompatibility of SHP_MBG_Sr

The fibroblast cell line L929 was selected to evaluate the biocompatibility of SHP_MBG_Sr. Cells were cultured at 37 °C in a humidified incubator equilibrated with 5% CO_2_ after the addition of an experimental cell culture medium (BIOCHROM KG, Berlin, Germany), prepared with Minimum Eagle’s Medium without L-glutamine, 10% fetal bovine serum, streptomycin (100 g L^−1^), penicillin (100 U mL^−1^) and 2 mmol L^−1^ L-glutamine, and they were placed in 250 mL plastic culture flask (Corning TM, Corning, NY, USA).

Cells were collected and then confluenced by means of a sterile trypsin-EDTA solution (0.5 g L^−1^ trypsin, 0.2 g L^−1^ EDTA in normal phosphate-buffered saline, pH 7.4), re-suspended in the experimental cell culture medium and diluted to 1 × 10^5^ cells mL^−1^. A preliminary qualitative assessment of the cell growth under the Transwell^®^ was conducted through optical imaging.

Cell viability tests were performed in a Transwell^®^ permeable insert. Briefly, fibroblast cells were seeded on the polystyrene plate below the Transwell^®^ insert containing 0.2 g of SHP_MBG_Sr, and after 72 h of incubation, cell viability was evaluated through an MTT assay. This assay allows assessing of the possible toxic effect of dissolution products and released ions on cells by evaluating the reduction of the mitochondrial succinate dehydrogenese (SDH) enzyme activity, normally involved in the citric acid cycle. Specifically, cells were incubated with 1 mg mL^−1^ solution of soluble tetrazolium salt (3-(4,5-dimethylthiazol-2yl)-2,5 diphenyl tetrazolium bromide) for 2 h at 37 °C. The SDH is supposed to cause the transformation of tetrazolium salts into a yellow soluble substance first and then into a blue water-insoluble product, the formazan precipitate. The amount of precipitate product is directly correlated to the enzyme activity and, consequently, to the number of metabolically active cells. In order to evaluate the active cells, the formazan precipitate was dissolved with dimethylsulphoxide and spectrophotometrically measured at a wavelength of 570 nm, providing an optical density (OD) value. Cells grown on a polystyrene plate were used as a negative control, while cells grown with the addition of 20 µL of a solution of 0.08 mg mL^−1^ of Sodium nitroprusside (NPS) were used as the positive one.

#### 2.4.2. Osteogenic Response to SHP_MBG_Sr

To evaluate the osteogenic response, osteoblast-like SAOS2 cells were selected and cultured at 37 °C in a humidified incubator equilibrated with 5% CO_2_. Cell suspension was obtained by adding 2 mL of a sterile 0.5% Trypsin-EDTA solution (GIBCO by Life Technologiey, ref. 15400-054, Thermoscentific, Watham, MA, USA) to a 250 mL cell culture flask (CorningTM), re-suspended in the experimental cell culture medium and diluted to 1.45 × 10^5^ cells mL^−1^.

Specifically, 5 mL of the cell suspension were seeded onto 24-well tissue culture polystyrene plates (FalconTM), provided with the Transwell^®^ insert, filled with 0.2 g of SHP_MBG_Sr. After 72 h and 7 days of incubation, the expression of GAPDH, COLL1a1, RANKL, SPARC, OPG and ALPL genes as cell differentiation markers was evaluated using the real-time reverse transcription polymerase chain reaction (qRT-PCR) (Applied Biosystems Assay’s ID: Hs00266705_g1, Hs00164004_m1, Hs00234160_m1, Hs00243519_m1, Hs00900358_m1, Hs01029144_m1, respectively).

The RNA from SAOS2 cells was firstly isolated using the Maxwell^®^ RSC simply RNA Cells Kit (Promega), by following the manufacturer’s instructions, and then reverse transcribed by the High-Capacity cDNA Reverse Transcription Kit (Applied Biosystems) and quantified before starting the RT-PCR using the Quantifluor system kit (Promega). The real-time PCR was performed in the Applied Biosystems StepOne Plus instrument (Applied Biosystems). The content of cDNA samples was normalized by using the comparative threshold cycle (ΔΔCt) method.

### 2.5. N-Acetylcysteine Loading into MBG_Sr

N-Acetylcysteine (NAC) was loaded into MBG_Sr (MBG_Sr_NAC) through the incipient wetness method (IW) by following a procedure reported in the literature [10,28]. In brief, 100 mg of MBGs were impregnated several times by dropping consecutive 100 µL aliquots of an ethanol-based NAC solution (concentration of 30 mg mL^−1^) onto the MBG_Sr powders at RT. After each impregnation, ethanol was evaporated at 50 °C for 10 min, and the dried powder was mixed with a spatula. The procedure consisted of the consecutive impregnation with six aliquots of 100 µL to allow the complete filling of the internal mesopores. Lastly, the obtained powders were dried at 50 °C overnight.

#### Characterization of MBG_Sr Loaded with NAC

Field-Emission Scanning Electron Microscopy (FE-SEM) (ZEISS MERLIN instrument—Oberkochen, Germany) was conducted to assess both the morphology and particle size of the MBG_Sr_NAC sample. To perform the observations, the MBG_Sr_NAC sample was dispersed directly onto the double face carbon tape placed on a sample stub and then coated with a Cr layer.

Nitrogen adsorption–desorption isotherms were measured by using an adsorption analyzer ASAP2020 Micromeritics (ASAP 2020 Plus Physisorption, Norcross, GA, USA) at a temperature of –196 °C. Firstly, NAC-loaded samples were outgassed for 5 h at 37 °C, in order to avoid the drug degradation. The Brunauer–Emmett–Teller (BET) equation was applied to calculate the specific surface area (SSA_BET_) from the adsorption data (relative pressures 0.04–0.2). On the other hand, the pore size distribution was evaluated through the DFT method (density functional theory) using the NLDFT kernel of equilibrium isotherms (desorption branch).

A Thermo-Gravimetric Analysis (TGA) of the samples was performed on a TG 209 F1 Libra instrument from Netzsch (Selb, Germany) over a range of 25–600° C under air flux at a heating rate of 10 °C min^−1^. The NAC drug content was the calculated from the weight loss between 200 and 600° C, subtracting the weight loss in the same range of temperature obtained for MBG_Sr alone (before NAC loading) ascribed to the surface silanol condensation.

The Fourier Transformed Infrared (FT-IR) spectra of the NAC-loaded samples were collected on a Bruker Equinox 55 spectrometer (Bruker, Billerica, MA, USA) over a range of wavenumbers from 4000 to 400 cm^−1^ (resolution 2 cm^−1^).

In order to assess the amorphous state of the incorporated NAC, *X*-ray patterns were collected using an X’Pert PRO, PANalytical instrument (X’Pert PRO, PANalytical, Almelo, The Netherlands) (CuK radiation at 40 kV and 40 mA). Data were obtained from 10° to 80° (diffraction angle 2θ) at a step size of 0.01° and a scan step time of 60 s.

Finally, to evaluate the Differential Scanning Calorimetry (DSC), NAC-loaded samples were heated from 37 °C to 200 °C with a heating rate of 10 °C min^−1^ under N_2_ flux, by using a DSC 204 F1 Phoenix (Netzsch) instrument (Selb, Germany).

### 2.6. Co-Release of Sr^2+^ Ion and NAC

#### 2.6.1. Sr^2+^ Ions/NAC Release Tests from MBGs

The ability of the MBG_Sr_NAC to release Sr^2+^ ions and NAC was investigated by following the procedure described in the literature [10,29].

The concentration of the released Sr^2+^ ions was evaluated by soaking the powders in Tris HCl buffer at a concentration of 250 µg mL^−1^. More in detail, in this experiment, 5 mg of powder were suspended in 20 mL of buffer and maintained at 37 °C in an orbital shaker (Excella E24, Eppendorf) with an agitation rate of 150 rpm for up to 14 days. At each selected time point (3 h, 24 h, 3 days, 7 days and 14 days), the suspension was centrifuged at 10,000 rpm for 5 min by using a Hermle Labortechnik Z326, and half of the supernatant was collected to quantify the concentration of the ions after appropriate dilutions by using the Inductively Coupled Plasma Atomic Emission Spectrometry (ICP-AES) technique. In order to maintain the volume of the release medium constant, at each time point, the removed supernatant was replaced by the same volume of fresh buffer solution. The release experiments were carried out in triplicate.

In order to express the results in terms of released percentage and to evaluate the total amount of ions incorporated into the MBGs during the synthesis, the powders were dissolved in a mixture of nitric and hydrofluoric acids (0.5 mL of HNO_3_ and 2 mL of HF for 10 mg of powder), and the resulting solutions were measured via ICP analysis. Each experiment was performed three times for each sample, and data are presented as means ± standard deviations.

The ability of the NAC-loaded MBG samples to release the incorporated drug was investigated by soaking the particles in Tris-HCl. More in detail, the concentration of drug released from the ion containing MBGs was evaluated by soaking the powders in Tris HCl buffer (Trizma, Sigma Aldrich), 0.1 M, pH 7.4, at a concentration of 20 mg mL^−1^. In particular, the powder (40 mg) was suspended in 2 mL of the buffer and kept in agitation (150 rpm) for 24 h at 37 °C in an orbital shaker (Excella E24, Eppendorf). After 1 h, 3 h, 20 h, 24 h and 48 h, the suspension was centrifuged at 10,000 rpm for 5 min by using a Hermle Labortechnik Z326, the total volume of supernatant was collected and the same volume of fresh buffer solution was finally added to maintain the volume of the release medium constant. The release experiments were carried out in triplicate. The concentration of the drug was measured by a Shimadzu SLC 40 High-Performance Liquid Chromatography (HPLC) equipped with the SPD-M40 diode array (8 technical replicates were analyzed) (Shimadzu, Kyoto, Japan). The released media were filtered through 0.2 µm cellulose acetate filters and analyzed at 30 °C using the Kyatech HiQSil C18 column from KyaTech. The mobile phase, composed by phosphate buffer 90% and methanol 10%, was used in the isocratic method, at a flow rate of 0.8 mL min^−1^ and a total run time of 10 min. The injection volume was 10 µL, and the main peak of NAC was identified at 198 nm. The drug content was finally quantified with respect to a calibration curve based on standards with a concentration ranging between 0.06 and 0.5 mg mL^−1^. The collected extracts were also characterized by ICP to measure the concentration of the released strontium ions.

#### 2.6.2. Incorporation of MBG_Sr_NAC into SHP Hydrogel

The hydrogel formulation based on SHP incorporating MBG_Sr_NAC (SHP_MBG_Sr_NAC) was prepared and characterized as reported in Section 2.3.1 and Section 2.3.2, respectively.

#### 2.6.3. Sr^2+^ Ions/NAC Co-Release Test from SHP_MBG_Sr_NAC

To evaluate both the ion/drug release from SHP_MBG_Sr_NAC, samples were firstly incubated at 37 °C for 15 min to allow their complete gelation; then, 1 mL of Trizma^®^ as a release medium was added to each sample, and at predefined time points (1 h, 3 h, 5 h, 1 d, 2 d, 3 d, 4 d, 8 d, 10 d and 14 d incubation time), the supernatant was collected and completely refreshed. The release of the drug was evaluated by using HPLC, according to the protocol described in Section 2.6.1. The collected extracts were also characterized by ICP to measure the concentration of the released strontium ions.

### 2.7. Statistical Analysis

Tube inverting and swelling/dissolution tests were conducted in triplicate, and results are expressed as mean ± standard deviation. The statistical analyses were performed using the GraphPad *t*-Test calculator (https://www.graphpad.com/quickcalcs/ttest1/, accessed on 1 May 2022). A *t*-Test analysis with a 95% confidence interval was used for comparisons.

## 3. Results and Discussion

### 3.1. Thermosensitive Injectable SHP Hydrogel Containing MBG_Sr (SHP_MBG_Sr)

Sr-substituted MBGs alone are not exploitable as long-term drug delivery systems for bone-healing applications, due to the strong burst release of Sr^2+^ ions once in contact with body fluids and the difficulties related to their administration and keeping in place at the pathological site (i.e., the bone fracture cavity). Therefore, their combination with an ad hoc developed hydrogel able to modulate the release kinetics and/or to act as a vehicle phase to the pathological site has been investigated. In particular, Sr-substituted MBGs have been incorporated into an injectable SHP-based thermosensitive hydrogel, and the sol-to-gel transition, injectability and stability of the resulting formulation (SHP_MBG_Sr) were fully investigated.

#### 3.1.1. Structural Characterization of NHP and SHP Polymers

**Figure 2:**
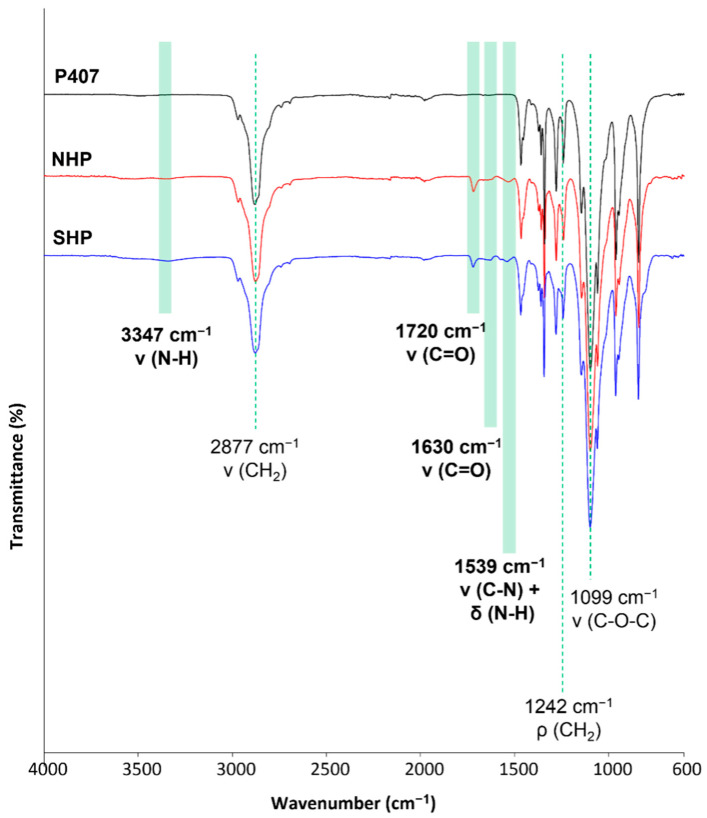
ATR-FTIR spectra of P407 (black), NHP (red) and SHP (blue). Light-green bars in the figure highlight the newly appeared peaks in the PEU spectra compared to the P407 one.

The successful synthesis of a high molecular weight poly(ether urethane) containing P407 as a building block was proven by ATR-FTIR spectroscopy and SEC analyses. In this regard, Figure 2 compares the ATR-FTIR spectra of the macrodiol P407 (black continuous line) and the as-synthesized NHP poly(ether urethane) (red continuous line). Light-green bars in the figure highlight the newly appeared peaks in the NHP spectrum that can be ascribed to the formation of urethane bonds during the synthesis: the absorption band at 3347 cm^−1^ due to the stretching vibration of N-H groups, the signals at 1720 and 1630 cm^−1^ produced by the stretching vibration of newly formed carbonyl groups and the peak at 1539 cm^−1^ ascribable to the simultaneous vibration of C-N (stretching) and N-H (bending) bonds. Absorption peaks due to the vibration of the -CH_2_ groups present along the PEU backbone appeared at 2877 cm^−1^ (stretching) and 1242 cm^−1^ (rocking), while the signal at 1099 cm^−1^ can be attributed to the asymmetric stretching of the -CH_2_-O-CH_2_- units present in the PEO blocks of P407.

The absence of signals at ca. 2200 cm^−1^ demonstrated the complete conversion of -N=C=O groups. Figure 2 also reports the registered ATR-FTIR spectrum of SHP poly(ether urethane) (blue continuous line), which did not exhibit any difference compared to NHP, thus demonstrating that the deprotection protocol did not alter the chemical structure of the PEU backbone. In addition, the SHP spectrum did not exhibit any signal due to the presence of residual chloroform or TFA molecules [27]. The absence of chemical degradation induced by the deprotection protocol was further demonstrated by SEC analyses, as NHP and SHP samples showed similar molecular weight distribution profiles (data not reported). The Number Average Molecular Weight values of NHP and SHP were estimated to be 64,500 Da (D = 1.87) and 65,000 Da (D = 1.83), respectively.

Lastly, the successful exposure of -NH_2_ groups along SHP polymeric chains was proven by adapting the colorimetric ninhydrin assay, usually used for amino acids and proteins, to synthetic polymers. NHP and SHP PEUs were both subjected to the assay, resulting in yellowish and blue/purple samples, respectively, meaning that in the former, no reaction between amino groups and ninhydrin molecules occurred. Differently, the dark blue/purple color of SHP samples directly proved the presence of amino groups producing the color transition of the reagents from yellowish to blue/purple. The number of exposed -NH_2_ groups along the SHP backbone was finally quantified through the UV-Vis spectroscopic analysis of the samples, resulting to be 2.3 × 10^17^ ± 4.9 × 10^16^ units/g by referring to a calibration curve based on glycine standards. The obtained result slightly differed from the one achieved on the same poly(ether urethane) through a different colorimetric assay for amine quantification, i.e., the Orange II sodium salt assay [27]. Such discrepancy can be attributed to differences between the adopted protocols for amino group quantification and the different working principle of the two assays. Indeed, the Orange II sodium salt assay exploits electrostatic interactions to conjugate amino groups and Orange II sodium salt molecules, whereas the ninhydrin assay exploits a redox reaction releasing ammonia (one of the products of the reaction occurring between ninhydrin and amino groups), which later reacts with a ninhydrin molecule, resulting in a colored complex called Ruhemann’s purple.

#### 3.1.2. Sol-to-Gel Transition, Injectability and Stability of SHP_MBG_Sr

The tube inverting test was conducted in temperature ramp and isothermal conditions at 37 °C to estimate SHP_MBG_Sr’s gelation temperature and gelation time in physiological conditions, respectively. Purely SHP hydrogels with 15% *w/v* concentration were also characterized for comparison. The gelation temperature of SHP and SHP_MBG_Sr sol-gel systems was estimated to be 28.3 ± 0.6 °C and 29.3 ± 0.6 °C, respectively. The addition of the MBGs proved to slightly increase the sol-to-gel transition temperature of SHP hydrogels. In agreement with our previous findings on similar hydrogels, [7,10] the MBGs embedded into the sol-gel system worked as defects within the hydrogel network during polymeric chain progressive arrangement into micelles and micellar aggregates and their assembly into a gel network. With regard to gelation time in physiological-like conditions, it slightly increased from 4.3 ± 0.6 min for the SHP hydrogel to 6.7 ± 0.6 min upon MBG_Sr loading at 20 mg mL^−1^ concentration. Overall, the encapsulation of MBG_Sr within the SHP hydrogel did not have detrimental effects on its sol-to-gel transition potential and timing [7,10,27].

SHP and SHP_MBG_Sr formulations were qualitatively tested in terms of injectability at 5, 25 and 37 °C through G22, G18 and G14 needles. The results of this qualitative evaluation are summarized in Table 1, where green and red colors identify injectable and not-injectable formulations under particular temperature/needle combinations. None of the potential users that tested injectability reported difficulty in formulation extrusion at 5 °C, irrespective of the considered needle. Differently, with increasing temperature up to 25 °C, both SHP and SHP_MBG_Sr formulations started to undergo a temperature-driven sol-to-gel transition that resulted in the impossibility to inject them through the smallest tested needle, namely the G22 needle, which exhibits an internal diameter of 413 μm. Instead, easy injectability was retained through both G18 and G14 needles. The temperature increases up to 37 °C did not induce any additional change in terms of injectability with respect to 25 °C, although it corresponded to a further organization of the formulations into more developed gel networks. No differences were observed in terms of injectability between SHP and SHP_MBG_Sr sol-gel systems, suggesting that the embedding of the MBGs within the polymeric matrix did not significantly alter the capability of the formulation to be manually extruded through different needles in the sol, semi-gel and gel state. The observed behaviour of SHP and SHP_MBG_Sr sol-gel systems is in agreement with our observations for similar formulations based on PEUs and inorganic particles [7,10,27].

**Figure 3:**
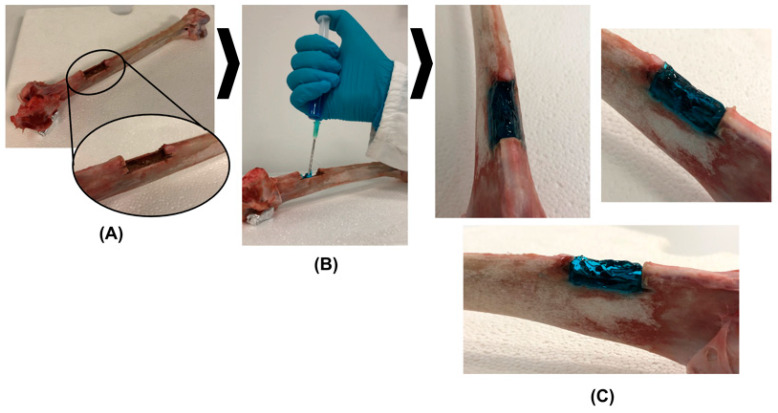
Qualitative ex vivo evaluation of SHP_MBG_Sr injectability in a turkey thigh bone with a manually-created defect in the diaphysis (**A**). The formulation exhibited easy injectability through a G18 needle (**B**) and the capability to perfectly fill the bone cavity while retaining its shape upon quick sol-to-gel transition (**C**).

SHP_MBG_Sr injectability was also ex vivo tested using a turkey thigh bone kept at 37 °C (Figure 3A). In detail, the SHP_MBG_Sr solution was injected through a G18 needle in the required amount to completely fill a previously created defect in the bone diaphysis. Then, the bone was again equilibrated at 37 °C to allow the complete sol-to-gel transition of SHP_MBG_Sr. The tested formulation proved to be easily injectable and able to undergo a quick sol-to-gel transition, which resulted in a relevant capability to retain the shape upon application (Figure 3B,C).

**Figure 4:**
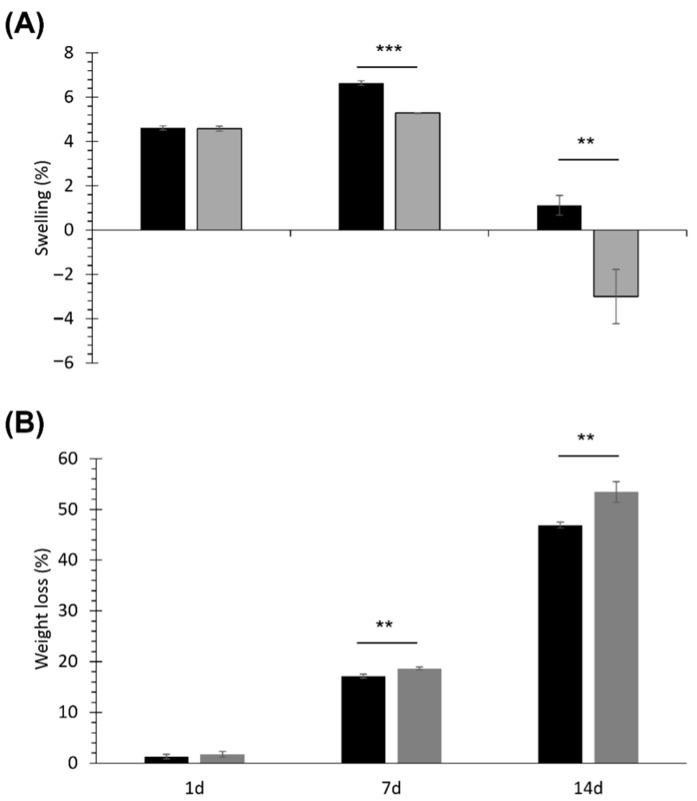
Percentage of swelling (**A**) and dried weight loss (**B**) for SHP (black bars) and SHP_MBG_Sr gels (grey bars). Significant results: *p* = 0.0001 (***) and *p* = 0.0055 (**) for swelling results on day 7 and 14, respectively; *p* = 0.0054 (**) and 0.0064 (**) for weight loss results on day 7 and 14, respectively.

Lastly, SHP and SHP_MBG_Sr gels were tested for their capability to absorb fluids and their stability during immersion in a watery medium. Figure 4 reports the trends of swelling and dried weight loss registered for both the investigated samples at 1, 7 and 14 days of immersion in a Tris HCl buffer solution (pH 7.4) at 37 °C. After 1 day of incubation in aqueous medium, SHP_MBG_Sr gels showed swelling and dried weight loss percentages similar to SHP samples (approx. 4.6% swelling and 1.5% weight loss). Differently, after 7 days of immersion, SHP_MBG_Sr gels exhibited a significantly different behaviour compared to SHP control gels: the swelling percentage of SHP_MBG_Sr was significantly lower (*p* = 0.0001) than the same parameter measured for SHP, because dissolution phenomena had started to prevail over fluid absorption in MBG-loaded formulations. As a matter of fact, on day 14, the swelling percentage of SHP_MBG_Sr assumed a negative value (around −3%), proving that dissolution had completely overwhelmed fluid absorption.

Conversely, for SHP gels, the swelling percentage at day 14 significantly decreased compared to 7 days of incubation (*p* = 0.0001), suggesting that for these samples, dissolution predominance over swelling was delayed compared to SHP_MBG_Sr gels. As a result, at both 7 and 14 days of incubation in aqueous medium, SHP gels showed a significantly lower weight loss percentage compared to SHP_MBG_Sr gels (*p* = 0.0054 and 0.0064 at 7 and 14 days, respectively). Thus, MBG encapsulation into SHP sol-gel systems significantly affected the stability of the resulting composite formulations in an aqueous medium [7,10], albeit with no detrimental effects (weight loss after 14 days of incubation was approx. 47 and 55% for SHP and SHP_MBG_Sr gels, respectively). However, differently from our recent results on PEU hydrogels embedded with MBGs releasing copper ions [10], in SHP_MBG_Sr gels, no chemical degradation was detected for the polymeric constituent of the samples up to 14 days of immersion in a Tris HCl buffer solution (SEC analyses revealed molecular weight and polydispersity index changes within the typical instrument error of approx. 10% [30]). Thus, the progressive release of strontium ions from the developed hybrid formulations did not induce any metal ion-mediated oxidation of PEU chains [31,32].

### 3.2. Biological Assessment of SHP_MBG_Sr

The following in vitro biological tests delt with the assessment of the biocompatibility of the developed hydrogel containing MBG_Sr and aimed to evaluate if the MBG_Sr were able to maintain their therapeutic effect by stimulating pre-osteoblastic cells after incorporation into the hydrogel [9]. The biocompatibility test on the system SHP_MBG_Sr was conducted using fibroblast cells (L929) and compared with a polystyrene plate as a negative control and with the polystyrene plate conditioned with sodium nitroprusside to induce cell death as a positive control.

In order to understand the contribution of each component of the system and highlight the role of the strontium ions, the cell viability after contact with the hydrogel alone (SHP) and the hydrogel containing both the MBG_Sr and the MBG without strontium (SHP_MBG_Sr and SHP_MBG, respectively) was evaluated.

**Figure 5:**
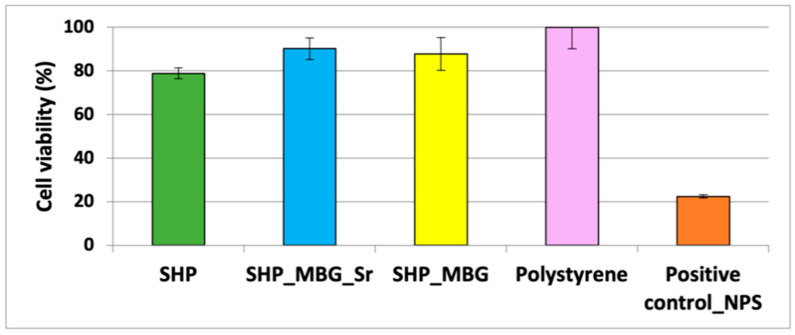
Quantification of cell viability through an MTT assay for SHP alone, SHP_MBG and SHP_MBG_Sr compared with polystyrene (negative control) and a positive control (polystyrene with 0.08 g mL^−1^ of NPS).

According to the international standard ISO 10993-5: 2009-Biological Evaluation of Medical Devices Tests for In vitro cytotoxicity, a cell viability percentage higher than 70% is the required minimum value for considering a material biocompatible. As reported in Figure 5, all the tested systems did not significantly alter the cell viability. Specifically, SHP alone showed a slight decrease in the cell viability, while both SHP_MBG_Sr and SHP_MBG showed excellent cytocompatibility, with a cell viability percentage ranging from 79% to 96%.

It is worth noticing that the cell viability of SHP_MBG_Sr resulted as higher compared with the SHP gel alone, suggesting a positive effect associated with the released ionic extracts and highlighting the beneficial role of the strontium ions.

In addition, microscope images of the cells, reported in Figure S3, further confirmed that all the tested formulations did not negatively affect the cell morphology.

**Figure 6:**
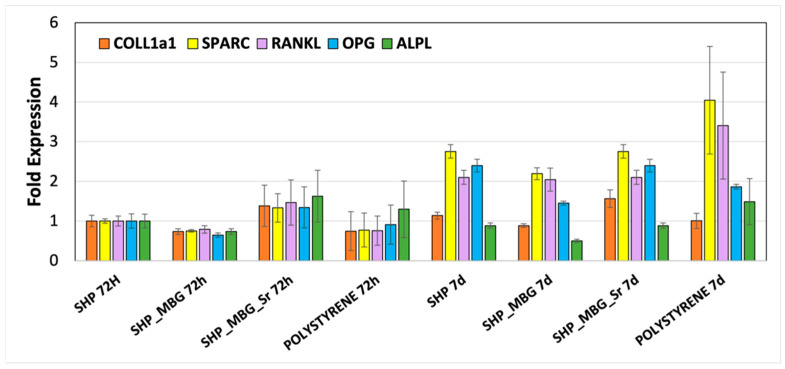
Gene expression of SAOS2 osteoblast-like cells at 72 h and 7 days of culture with SHP, SHP_MBG and SHP_MBG_Sr. Polystyrene was also tested as a control condition.

Pro-osteogenic properties of SHP_MBG_Sr were evaluated by performing the experiments with Transwell^®^ permeable inserts, able to prevent direct contact between the hydrogel and the cells while allowing one to elucidate the role of the released ions. The effect induced by the ionic extracts on the expression of pro-osteogenic genes was evaluated by a RT PCR analysis and is reported in Figure 6.

After 72 h of incubation, SHP_MBG_Sr showed a different gene expression compared to the SHP alone, with a RANKL/OPG ratio of 1.09 (values reported in Table 2). Overexpression of all of the five analyzed genes was observed, in particular, with a higher overexpression of the ALPL gene. This effect is specifically ascribed to the release of Sr^2+^ ions through the hydrogel, since SHP_MBG, on the contrary, showed a downregulation gene expression at 72 h. Compared to the time point at 72 h, the cells incubated with SHP_MBG_Sr for 7 days (Table 2) showed an overexpression of COLL1a1, SPARC, RANKL and OPG, with a RANKL/OPG ratio of 0.88 in favor of OPG which resulted over-expressed. Interestingly, at 7 days, this ratio switched in favor of RANKL in the SHP_MBG and polystyrene cultured cells, while it remained almost unchanged in the presence of SHP alone. In particular, this ratio was 1.83 for cells grown on polystyrene plates, 0.88 on SHP alone and 1.41 on SHP_MBG. A material can be considered to exert a pro-osteogenic effect if it is able to promote the overexpression of the OPG gene, which is responsible for the pro-osteogenic stimulation. Thus, the ability of the SHP_MBG_Sr to induce the switch of the RANKL/OPG ratio in favor of OPG, as well as the overexpression of SPARC and COLL1a1 genes, could be ascribed to the effect of the released Sr ions, able to positively affect the gene expression after 7 days of incubation.

As reported for the MBG_Sr as such in our previous work [9], the switch in the RANKL/OPG ratio in favor of OPG expression after 7 days of incubation in the presence of ionic extracts from SHP_MBG_Sr (at variance with unsubstituted MBGs) suggests the inhibition of osteoclastogenesis, confirming the pro-osteogenic response induced by the released Sr^2+^ ions in an amount suitable to exert an in vitro therapeutic effect.

Overall, these preliminary in vitro results evidenced the biocompatibility of the developed SHP_MBG_Sr system, also confirming that the embedded MBG_Sr particles retained a pro-osteogenic effect induced by the diffusion of Sr^2+^ ions throughout the hydrogel.

### 3.3. Morphological, Structural and Chemical Characterization of MBG_Sr_NAC

**Figure 7:**
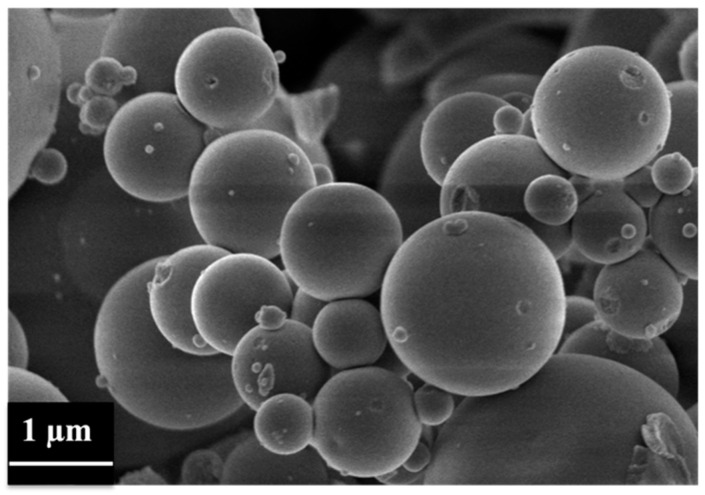
FE-SEM image of MBG_Sr_NAC.

Figure 7 shows the FE-SEM images of MBG_Sr_NAC, revealing microparticles with a size ranging between 1 and 5 µm. FE-SEM observation showed that the incorporation of NAC by the IW method did not significantly alter the particle morphology and size, which resulted very close to those reported for analogue samples before drug incorporation [6,9].

**Figure 8:**
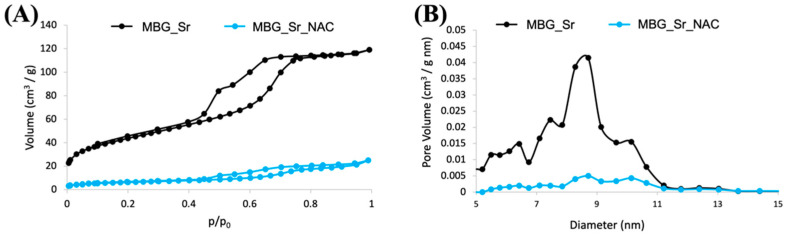
(**A**) N_2_ adsorption–desorption isotherm of MBG_Sr_NAC compared to the not-loaded sample (**A**) and (**B**) related DFT pore size distribution (**B**).

The N_2_ adsorption–desorption isotherms and the pore size distribution of MBG_Sr before and after drug loading are reported in Figure 8A,B, respectively. The isotherm of MBG_Sr was a type IV curve (Figure 8A), with an H1 hysteresis loop, typical of mesoporous materials with pores larger than 4 nm, and the pore size distribution showed pores with a size ranging between 8 and 11 nm, with a maximum distribution centered at around 9 nm (Figure 8B), allowing the easy diffusion and incorporation of NAC molecules by IW.

As shown in Figure 8 and reported in Table 3, the drug loading led to an appreciable modification of the adsorption–desorption isotherm (reduction of the adsorbed volume and presence of hysteresis loop) and a significant reduction of the pore volume. More in detail, MBG_Sr_NAC showed a drastic reduction in both SSA and pore volume, suggesting that most mesopores were fully filled with NAC, and a small fraction experienced size reduction and shape modification from cylinder to ink-bottle pores, as already noted previously in the literature by Hong et al. [33]. The drastic reduction of pore volume as a consequence of drug incorporation was confirmed by the almost full disappearance of the component centered at around 9 nm (Figure 8B).

**Figure 9:**
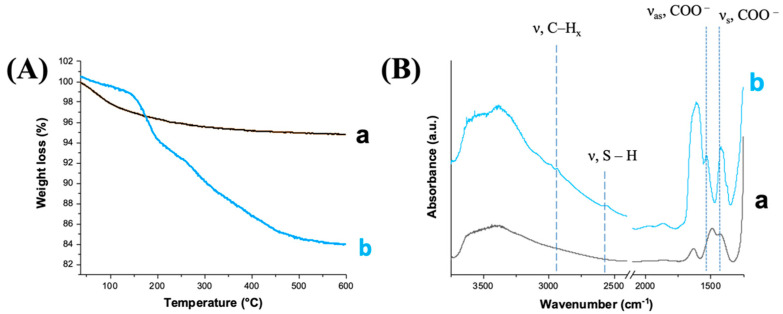
(**A**) TGA thermograms of (a) MBG_Sr and (b) MBG_Sr_NAC and (**B**) FT-IR spectra of (a) MBG_Sr and (b) MBG_Sr_NAC.

The total amount of loaded NAC was quantified by TGA analysis. The TGA thermogram of MBG_Sr_NAC (Figure 9A) exhibited a significant weight decrease between 200 and 400 °C, at variance with the TGA curve obtained for MBG_Sr, also analyzed as a reference. The observed weight loss is ascribable to the loss of NAC molecules [34], confined into the MBG framework by multiple intermolecular interactions (i.e., H-bonding, dispersive forces), as already reported in the literature for other incorporated drugs [35]. Based on the TGA analysis, the weight percentage of NAC loaded into MBG_Sr resulted as being 12% (*w*/*w*).

The FT-IR spectra of MBG_Sr and MBG_Sr_NAC are reported in Figure 9B. All samples showed the typical absorption bands (stretching vibration) of H-bonded hydroxyls in the range of 3750–3000 cm^−1^. Concerning the NAC-loaded sample, the FT-IR spectrum of MBG_Sr_NAC showed the typical bands associated to NAC: at 2935 cm^−1^, the absorption band was ascribed to C-H stretching modes, and at 1535 and 1430 cm^−1^, it was due to the asymmetric (ν_as_) and symmetric (ν_s_) stretching vibration of the carboxylate group COO^−^, respectively [35]. In addition, the typical band associated with the S-H stretching of the thiol groups appeared at 2565 cm^−1^, although it was slightly intense due to the intrinsic low extinction coefficient, further proving the successful incorporation of NAC into the MBG pores.

**Figure 10:**
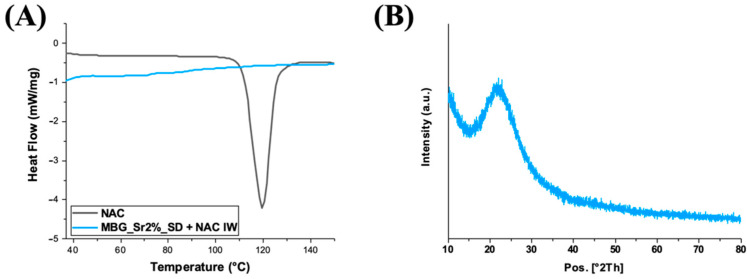
(**A**) DSC thermograms of NAC compared to MBG_Sr_NAC and (**B**) the XRD pattern of MBG_Sr_NAC.

As widely reported in the literature [36,37], the loading of the drug in the amorphous state could increase the drug dissolution rates and solubility. Hence, DSC and XRD analyses of NAC-loaded samples were conducted to prove the amorphous state of the drug and exclude the presence of large crystalline aggregates. Both the DSC thermograms of NAC, as such, and of MBG_Sr_NAC are reported in Figure 10A: the single endothermic melting peak at 120 °C, observed only for NAC, as such, corresponded to crystal phase melting. The absence of this peak in the thermogram related to the NAC-loaded sample confirmed the non-crystalline form of the drug adsorbed into the mesopores.

The amorphous state of the drug was further assessed by XRD analysis (Figure 10B). In accordance with DSC data, the XRD pattern of MBG_Sr_NAC powder evidenced that the re-crystallization of the drug did not occur inside the pores upon solvent evaporation during the incorporation process, and indeed, only a broad peak typical of amorphous materials was shown, without any reflection due to the crystalline drug.

### 3.4. Sr^2+^/NAC Co-Release from MBG_Sr_NAC

**Figure 11:**
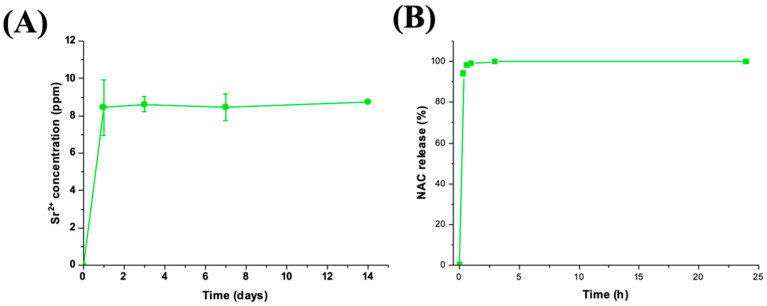
Released ppm of Sr^2+^ ions (**A**) and percentage of NAC released (**B**) from MBG_Sr_NAC in Tris-HCl.

The concentrations of Sr^2+^ ions released over time from MBG_Sr_NAC when soaked in Tris HCl medium are reported in Figure 11A,B. A burst release in the first 3 h of soaking followed by a stationary trend up to 14 days of incubation was observed, similarly to MBG_Sr [6,9], as a consequence of the very fast ionic exchange reactions occurring at the mesopore surface. The final strontium concentration after 3 h of soaking was 8.1 ppm for MBG_Sr_NAC, which represented the 100% of the amount initially incorporated into the sample, as assessed by the ICP analysis on acidic-digested MBG_Sr_NAC powder. The obtained results show that NAC loading within the porous structure did not significantly interfere with the capability to release Sr^2+^ ions through ion-exchange reactions. An N-Acetylcysteine release test was performed in Tris-HCl for up to 24 h, and Figure 11B shows the obtained release profile, evidencing that the total loaded amount of NAC was released in the first hour of soaking in the medium.

As already reported in the literature for other drugs, the weak interactions between the drug molecules and the MBG surface combined with the open porous structure, which facilitates the drug diffusion from the inner pores to the soaking medium, lead to the observed strong burst release [38,39].

### 3.5. Co-Release of NAC and Sr^2+^ Ions from SHP_MBG_Sr_NAC

Once assessed the sustained release of strontium ions from the hydrogel and the retained associated pro-osteogenic/anti-osteoclastogenic effect, the co-delivery of both strontium ions and NAC from the SHP_MBG_Sr_NAC hydrogel formulation was evaluated, with the aim to develop a multifunctional platform to be injected directly at the pathological site and able to simultaneously co-release ion/drug therapeutics.

**Figure 12:**
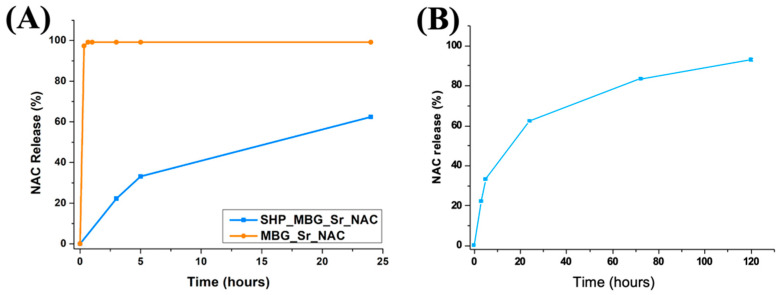
(**A**) Comparison among the NAC release profiles assessed from MBG_Sr_NAC and SHP_MBG_Sr_NAC up to 24 h observation time and (**B**) the NAC release profile from SHP_MBG_Sr_NAC assessed up to 7 days.

Figure 12A shows the NAC release profiles from MBG_Sr_NAC and SHP_MBG_Sr_NAC up to 24 h. As expected, MBG_Sr_NAC showed a burst NAC release profile, with the total amount of loaded NAC released in the first hour of incubation. By the comparison of the kinetics within the first 24 h of NAC release from MBG_Sr_NAC alone (orange curve) and from hydrogel-containing MBG_Sr_NAC (blue curve), the role exerted by the polymeric matrix in modulating the release profile of the drug is clearly highlighted, with a significant reduction in the initial burst release of about 80% in the first three hours and 40% at the end of the 24 h of observation.

Moreover, the NAC release from SHP_MBG_Sr_NAC was sustained over time, reaching a release percentage of 90% after 7 days of incubation (Figure 12B).

Some recent reports have highlighted the role of NAC in regulating cellular differentiation towards various cell phenotypes, inducing a dose-dependent promotion of the osteogenic differentiation of osteoblast-like cells [23,24,40] without toxicity or apoptosis. It is worthy to notice that the NAC released at 24 h from the SHP_MBG_Sr_NAC reached a concentration of around 5 mM, and at the end of the 7 days of soaking, it reached around 8 mM, falling within the concentration range proven in the literature to induce the activation of the differentiation of osteogenic lineages (from 2.5 mM to 10 mM [23]). In this perspective, the reported sustained release of NAC could guarantee the timely administration of the drug directly at the pathological site at the initial stage and during the first week of the healing process and positively affect the bone tissue’s healing.

**Figure 13:**
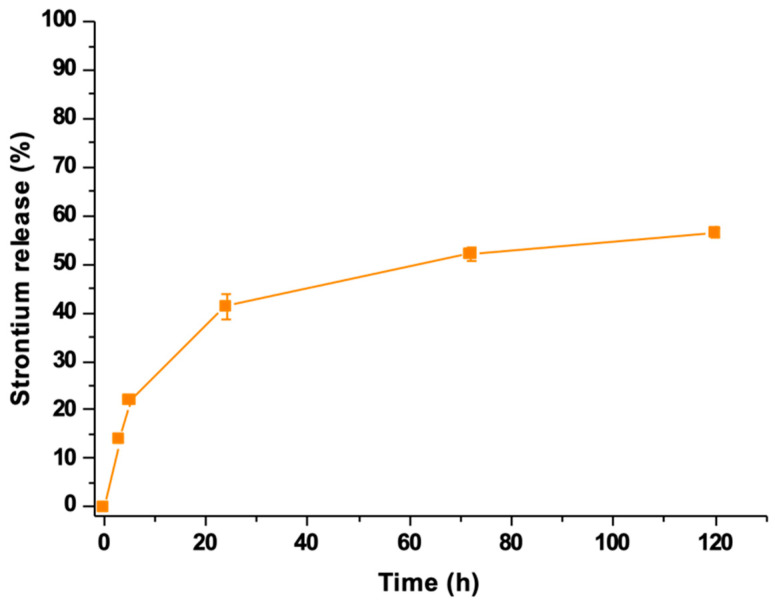
Sr^2+^ ions release profile from the SHP_MBG_Sr_NAC hydrogel.

The release profile of strontium ions from the developed hydrogel formulation was investigated to evidence potential hindering by the drug loaded into the pores. The observed release showed a trend similar to that assessed for N-Acetylcysteine (Figure 13). After 7 days of incubation in aqueous medium, the 56% of the strontium initially present in the MBG framework was released from SHP_MBG_Sr_NAC. The incorporation of MBG_Sr_NAC within the polymeric phase successfully decreased the undesirable initial burst release of Sr^2+^ species typically observed for particles as such. In fact, after 1 h of incubation in similar releasing conditions, SHP_MBG_Sr_NAC released an amount of strontium ions approximately 80% lower compared to the MBG_Sr_NAC particles alone, further confirming the role exerted by the hydrogel in modulating the release rate, as already observed for the NAC release.

## 4. Conclusions

In this work, a multifunctional injectable formulation for localized and long-term co-delivery of Sr^2+^ ions and NAC was successfully developed by incorporating strontium-containing MBG microspheres loaded with NAC into a thermosensitive polyurethane-based hydrogel, able to act as a vehicle phase at the site of the bone defect and to guarantee a sustained co-release of ions/drugs in situ.

The composition of the injectable hydrogel was tailored in order to improve both the gelation kinetics and the stability in an aqueous environment. The embedded MBG_Sr did not alter the ability of the developed formulation to undergo fast gelation in physiological conditions, and injectability was proven in a wide range of temperatures through different needles. The injectability was also ex vivo assessed using a turkey thigh bone kept at 37 °C, revealing that the formulation is easily injectable and able to undergo a quick sol-to-gel transition, with a relevant capability to retain shape upon application and a high potential for the final clinical translation. The biological assessment revealed the full biocompatibility of the hydrogel-based system and the retained ability to promote the overexpression of pro-osteogenic genes, due to the release of appropriate strontium ion concentrations. The incorporation into the hydrogel proved to play a key role in the modulation of the ion/drug release from the incorporated carriers, allowing the co-release of Sr^2+^ ions and NAC with sustained and prolonged kinetics (monitored up to 120 h) with respect to MBG_Sr particles, as such, which fully deliver their cargo in the first hours of soaking. The final concentration of released NAC from the developed hydrogel formulation (*ca* 8 mM) was aligned to the value reported in the literature able to positively affect the bone healing process by inducing the activation of the differentiation of osteogenic lineages.

In addition, the developed platform showed a high degree of versatility, since the type of therapeutics and their final released concentration could be easily tuned depending on the required clinical target, showing great potential as in situ injectable delivery systems for the treatment of the compromised regeneration of soft (e.g., chronic wounds) and hard tissues (e.g., delayed bone healing).

## Figures and Tables

**Table 1:** Qualitative evaluation of injectability for SHP and SHP_MBG_Sr sol-gel systems. Tests were conducted by different users at three temperatures (i.e., 5, 25 and 37 °C) through three different needles (G22 Φ_int_ = 0.413 mm, G18 Φ_int_ = 0.838 mm, G14 Φ_int_ = 1.60 mm).

		SHP	SHP_MBG_Sr
5 °C	G22	Injectable	
	G18	Injectable	
	G14	Injectable	
25 °C	G22	Non- Injectable	
	G18	Injectable	
	G14	Injectable	
37 °C	G22	Non- Injectable	
	G18	Injectable	
	G14	Injectable	

**Table 2:** RANKL/OPG ratio after 72 h and 7 days of SAOS2 cell culture.

Sample	RANKL/OPG Ratio
**72 h**	
**SHP**	1.00
**SHP_MBG**	1.22
**SHP_MBG_Sr**	1.09
**Polystyrene**	0.83
**7 days**	
**SHP**	0.88
**SHP_MBG**	1.41
**SHP_MBG_Sr**	0.88
**Polystyrene**	1.83

**Table 3:** SSA and pore volume of MBG_Sr_NAC compared to the MBG_Sr.

Sample	SSA_BET_ (cm^2^ g^−1^)	Pore Volume (cm^3^ g^−1^)
MBG_Sr	156	0.18
MBG_Sr_NAC	22	0.03

## Data Availability

Not applicable.

## Supplementary Materials

The following supporting information can be downloaded at https://www.mdpi.com/article/10.3390/pharmaceutics14091890/s1. Figure S1: Chemical structures of poly(urethane) building blocks and the scheme of the protocol adopted for NHP poly(urethane) synthesis; Figure S2: Scheme of the Boc deprotection protocol performed on NHP to obtain SHP; Figure S3: Optical images of L929 fibroblast cells grown under the insert containing SHP, SHP_MBG and SHP_MBG_Sr for 72h; images of the positive and negative control are also reported. Scale bar: 100 µm.
