# Supplementary material for: Mesoporous Bioactive Glasses Incorporated into an Injectable Thermosensitive Hydrogel for Sustained Co-Release of Sr2+ Ions and N-Acetylcysteine
Source: Pharmaceutics. 2022 Sep 7;14(9):1890. doi: 10.3390/pharmaceutics14091890 (PMC9504849; doi:10.3390/pharmaceutics14091890)
Supplement: Supplementary file 1 [file pharmaceutics-14-01890-s001.zip › pharmaceutics-1854610-supplementary.pdf]

Mesoporous bioactive glasses incorporated into an injectable thermosensitive hydrogel for sustained co-release of Sr^{2+} ions and *N*-acetylcysteine

Carlotta Pontremoli[1,2], Monica Boffito[3], Rossella Laurano[3], Giorgio Iviglia[4], Elisa Torre[4], Clara Cassinelli[4], Marco Morra[4], Gianluca Ciardelli[3], Chiara Vitale-Brovarone[1],
Sonia Fiorilli[1*]

Supporting information

Table of content

S1: Chemical structures of poly(urethane) building blocks and scheme of the protocol adopted for NHP poly(urethane) synthesis.

S2: Scheme of the Boc deprotection protocol performed on NHP to obtain SHP.

S3: Optical images of L929 fibroblast cells grown under the insert containing SHP, SHP_MBG and SHP_MBG_Sr for 72h; images of positive and negative control are also reported. Scale bar: 100 μm.

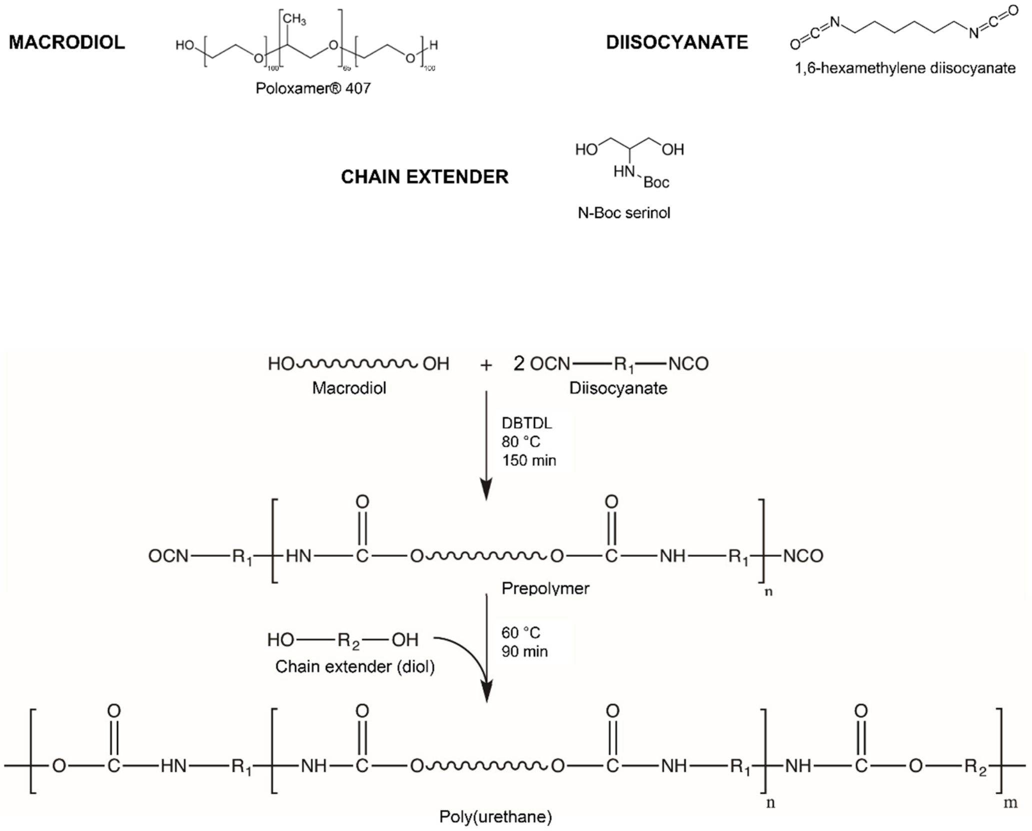

Figure S1 Chemical structures of poly(urethane) building blocks and scheme of the protocol adopted for NHP poly(urethane) synthesis.

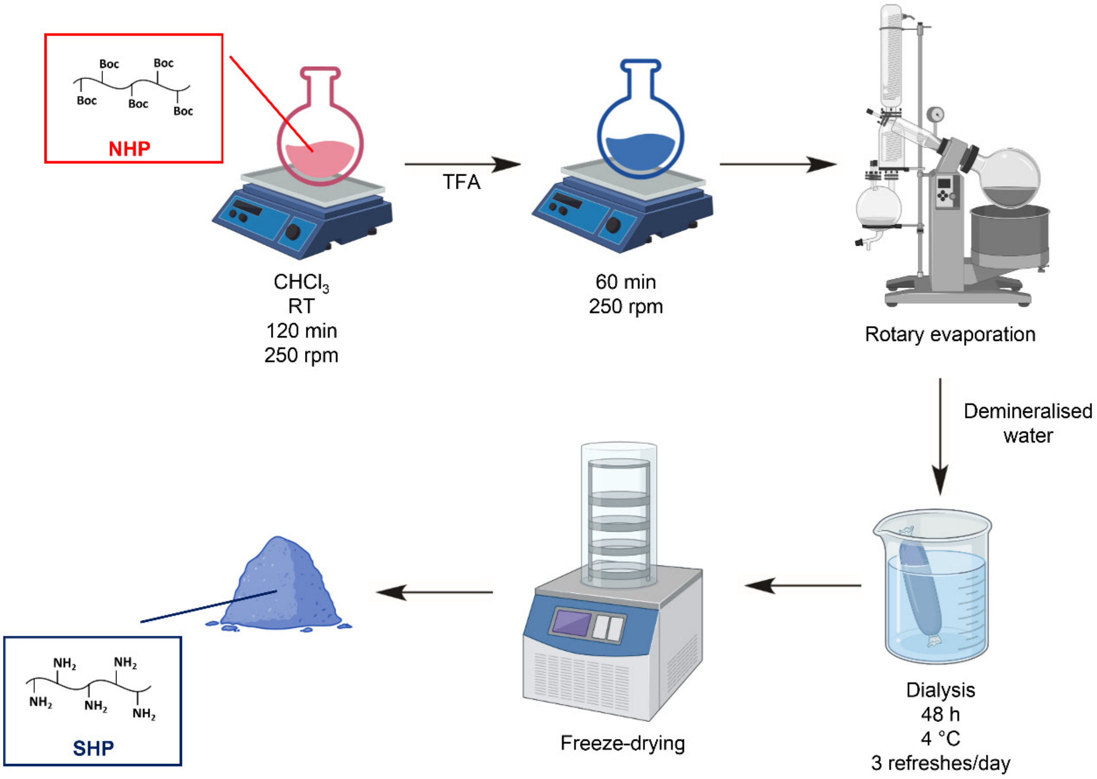

Figure S2 Scheme of the Boc deprotection protocol performed on NHP to obtain SHP.

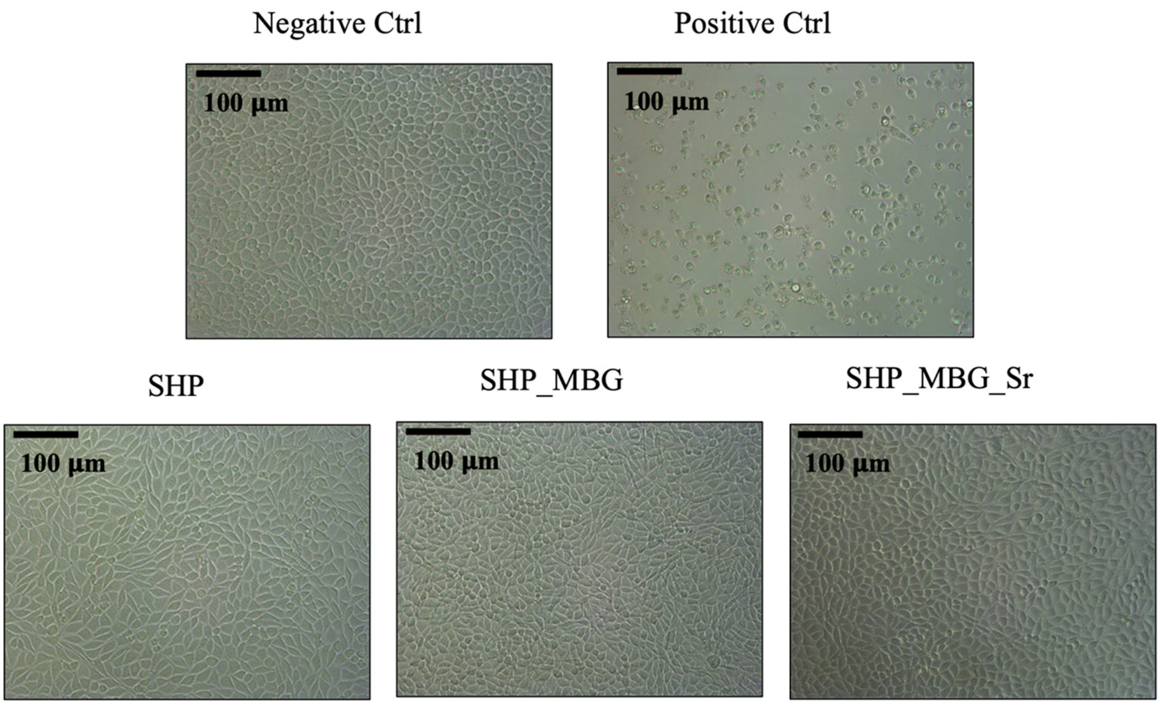

Figure S3 Optical images of L929 fibroblast cells grown under the insert containing SHP, SHP_MBG and SHP_MBG_Sr for 72h; images of positive and negative control are also reported. Scale bar: 100 µm.
